# Impact of duodenum-preserving pancreatic head resection and pancreatoduodenectomy on postoperative diarrhea: insights from trial sequential analysis

**DOI:** 10.1097/JS9.0000000000001525

**Published:** 2024-05-03

**Authors:** Dan-Na Xie, Bao-Lin Qian

**Affiliations:** a The First Clinical Medical College of Lanzhou University; b Department of Hepatobiliary Surgery, Affiliated Hospital of Southwest Medical University, Luzhou, People’s Republic of China

*Dear Editor,*

We commend the recent work of Yin *et al*.^1^ for their rigorous and detailed systematic review of seven clinical trials, focusing on the debate over whether the duodenum-preserving pancreatic head resection (DPPHR) has an advantage over pancreatoduodenectomy (PD) in terms of long-term benefits. The efforts made by the authors will offer potential benefits in reducing complications and promoting patient recovery. The authors found that there were no significant differences in quality of life (QOL) scores between the DPPHR and the PD (Standard Mean Difference: 0.21, 95% CI: −0.05 to 0.46, *P*=0.109, *I* ^2^=70%). However, the DPPHR had advantages over the PD in terms of longer OS (overall survival) times (hazard ratio: 0.59, 95% CI: 0.44–0.77, *P*<0.001, *I* ^2^=0%) and lower symptom scores for diarrhea (Standard Mean Difference: −0.36, 95% CI: −0.66 to −0.06, *P*=0.020, *I* ^2^=64%). These outcomes suggest that the DPPHR should be recommended over PD for the treatment of benign pancreatic diseases and low-grade malignant tumors.

Diarrhea is a common complication after pancreatectomy, and 39.0% of patients undergoing PD have refractory diarrhea after surgery^2^. Refractory diarrhea is often associated with pancreatic exocrine function and peripheral nerve injury, which seriously affects the expected QOL of patients^3,4^. Here, the authors found that DPPHR can effectively improve the symptoms of diarrhea in patients compared with PD, which has great benefits for the long-term prognosis of patients. It’s worth noting that the current evidence to support DPPHR to improve diarrhea symptoms may be insufficient. To examine the robustness of evidence, we further analyzed the raw data from the original meta-analyses by performing trial sequential analysis (TSA), a method used to evaluate the cumulative evidence in meta-analyses^5^. Although the *Z*-curve exceeds the conventional statistical significance boundary, it does not cross the trial sequential monitoring boundary (Fig. 1). This suggests that there is insufficient evidence that DPPHR surgery improves diarrhea symptoms in patients. Nevertheless, the study of Yin *et al*.^1^ still made significant contributions to preventing postoperative diarrhea and improving the long-term prognosis of patients. Our analysis suggests that additional studies are needed to determine whether DPPHR is different from PD in terms of diarrhea symptoms.

**Figure 1:**
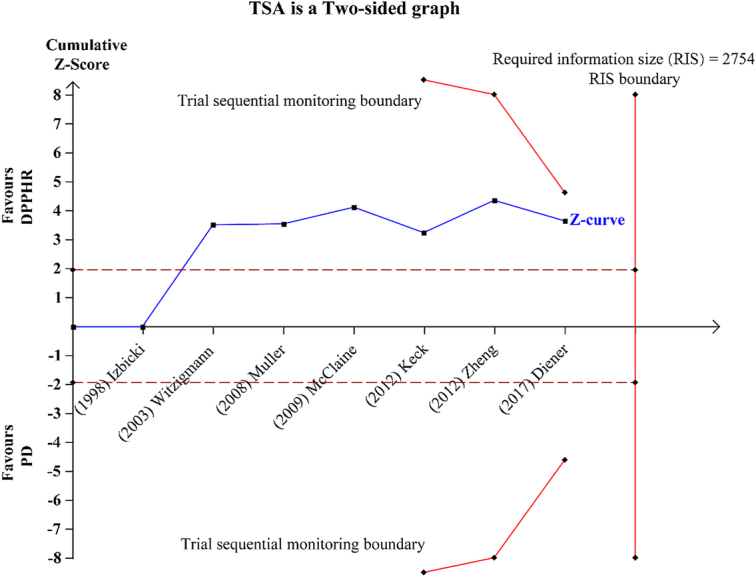
Trial sequential analysis was conducted to evaluate the difference between the DPPHR and PD in postoperative symptoms of diarrhea. The blue shows the *Z*-curve, the red dotted line shows the conventional statistical significance boundary and the solid red line shows the trial sequential monitoring boundary. DPPHR, duodenum-preserving pancreatic head resection; PD, pancreatoduodenectomy; TSA, trial sequential analysis, RIS, required information size.

## Ethical approval

Not applicable.

## Consent

Not applicable.

## Sources of funding

No external funding was received for this study.

## Author contribution

D.-N.X.: data curation, formal analysis, methodology, software, validation, writing – original draft, and writing – review and editing; B.-L.Q.: conceptualization, supervision, software, validation, writing – original draft, and writing –review and editing.

## Conflicts of interest disclosure

The authors declare no conflicts of interest.

## Research registration unique identifying number (UIN)

Not applicable.

## Guarantor

Bao-Lin Qian.

## Data availability statement

The datasets used and/or analyzed in the current study are available from the corresponding author upon reasonable request.

## Provenance and peer review

This paper was not invited.

## References

[1] Yin T Wen J Zhen T . Long-term quality of life between duodenum-preserving pancreatic head resection and pancreatoduodenectomy: a systematic review and meta-analysis. Int J Surg 2024;110:1139–1148. 38000055 10.1097/JS9.0000000000000879 PMC10871662

[2] Kuroki N Ono Y Sato T . Long-term outcome of patients with postoperative refractory diarrhea after tailored nerve plexus dissection around the major visceral arteries during pancreatoduodenectomy for pancreatic cancer. World J Surg 2022;46:1172–1182. 35119513 10.1007/s00268-022-06457-5

[3] Latenstein AEJ Blonk L Tjahjadi NS . Long-term quality of life and exocrine and endocrine insufficiency after pancreatic surgery: a multicenter, cross-sectional study. HPB (Oxford) 2021;23:1722–1731. 34001452 10.1016/j.hpb.2021.04.012

[4] Kato H Kamei K Suto H . Incidence and risk factors of nonalcoholic fatty liver disease after total pancreatectomy: a first multicenter prospective study in Japan. J Hepatobiliary Pancreat Sci 2022;29:428–438. 34863034 10.1002/jhbp.1093

[5] Hung KC Chuang MH Chen JY . Impact of intravenous vitamin C as a monotherapy on mortality risk in critically ill patients: a meta-analysis of randomized controlled trials with trial sequential analysis. Front Nutr 2023;10:1094757. 37051117 10.3389/fnut.2023.1094757 PMC10083893

